# Twenty years of dysuria in a patient with Addison’s disease: a case report

**DOI:** 10.4076/1757-1626-2-7995

**Published:** 2009-06-15

**Authors:** Gabriella N Nanci, Millard J Collier, Sheldon H Rose

**Affiliations:** Department of Internal Medicine, Atlanta Medical Center303 Parkway Drive NE, Atlanta, GA 30312USA

## Abstract

X-linked adrenoleukodystrophy is an X-linked recessive disorder affecting approximately 1 in 21,000 males, and is estimated to be the cause of adrenal insufficiency in approximately 35% of patients with idiopathic Addison’s disease. The disease is caused by defective beta-oxidation of fatty acids in peroxisomes that leads to elevated serum concentrations of very-long-chain saturated fatty acids. The accumulation causes a primary adrenal insufficiency and progressive neurological dysfunction. This article presents a case of X-linked adrenoleukodystrophy in its milder form, adrenomyeloneuropathy.

## Case presentation

A 45-year-old white male building contractor presents to a family practice clinic with symptoms of dysuria and pain on micturation. He has used cranberry tablets, with some relief. He has had this problem on and off for 20 years. He was recently treated him with doxycycline, which failed to help. He has no history of sexually transmitted diseases.

The patient’s history is significant for Addison’s disease. He was diagnosed at age 19 when he presented to a hospital with extreme weakness. He was placed on maintenance doses of hydrocortisone and fludrocortisone. The patient reports childhood illness that included difficulty running and a year of quarantine due to an unidentified infection. The patient complains of unsteadiness while walking and is concerned he might have adrenoleukodystrophy. He had previously requested a serum B12 test, which was normal. He feels short of breath at times and has lower back pain. The patient is also concerned about symptoms of irritability and depression.

His medication list includes hydrocortisone 20 mg daily, fludrocortisone 0.1 mg daily, escitalopram 10 mg daily, dehydroepiandrosterone (DHEA) 50 mg daily, ibuprofen 800 mg twice daily.

Both of the patient’s parents are living. The patient’s mother has coronary artery disease and Type II Diabetes. The patient is one of 10 siblings, 8 living, including one set of triplets and one set of twins. The patient is a fraternal twin. One of the brothers died unexpectedly two weeks after birth, another brother died of influenza at the age of eight. A third brother lives in an institution due to severe mental retardation. A fourth brother has pronounced ataxia.

On physical exam the patient appears well. He is 6′4′′;and 230 pounds. Blood Pressure 119/88, Pulse, 102. Lungs are clear. Abdomen is soft and non-tender. Hyperpigmentation is clearly observed, especially on the knees. His hair appears dry and sparse and eyelashes are minimal. There is diminished sensation and vibration sense in his feet. Mild ataxia is present and the patient walks with heals off the ground. Romberg is intact. Plantar reflex is extensor bilaterally, (Babinski sign).

Patient was treated with Ciprofloxacin for urethritis. Endocrinology and neurology consults were sought.

### Differential diagnosis

Polyglandular Endocrine Deficiency Syndrome, tuberculosis, Ectopic ACTH Syndrome, adrenomyeloneuropthy, multiple sclerosis, prostate disease, Addison’s Disease, and Triple A Syndrome, (achalasia-addisonianism-alacrima syndrome).

### Investigative results

**Table:** 

Urinalysis	
Appearance:	Clear yellow
Urobilinogen:	Normal
Glucose:	Negative
Ketone:	Negative
Bilirubin:	Negative
Protein:	30
Nitrite:	Negative
Leukocytes:	+
Blood:	Negative
pH:	5
Specific Gravity:	1.030
Urine Culture:	Negative

**Table:** 

Imaging
Chest X-ray: Normal
Brain MRI:
1. Negative brain. Variation of normal asymmetry of the right lateral ventricle.
2. Minor intrasellar cistern but essentially normal adenohypophysis as described.
3. Chronic pansinusitis

### Other tests

**Table:** 

Insulin-Like Growth Factor-1	133 In Range	(90-360 ng/ml)
Testosterone:	265 In Range	(241-827 NG/DL)
PSA:	1.02	(0-4.0 ng/ml)
ACTH	135 pg/ml High	(15-80 pg/ml)

**Table:** 

Fatty Acid Profile, Peroxisomal C22-C26:		
C22:0	69.2 In Range	(< OR = 96.3 UMOL/L)
Pristanic Acid	0.55 In Range	(< OR = 2.98 UMOL/L)
Phytanic Acid	3.73 In Range	(< OR = 9.88 UMOL/L)
Pristance Phytanic	0.15 In Range	(< OR = 0.39 Ratio)
C24:0	97.2 High	(< OR = 91.4 UMOL/L)
C26:0	4.06 High	(< OR = 1.30 UMOL/L)
C24:0/C22:0	1.40 High	(< OR = 1.39 Ratio)
C26:0/C22:0	0.058 High	(< OR = 0.023 Ratio)

## Discussion

The history, physical, and symptoms of our patient resulted in the consideration of several possibilities as is evident by our differential diagnosis list. However, elevated concentrations of C24, C26, as well as abnormally high C24/C22 and C26/C22 ratios are indicative of hemizygosity for X-linked adrenoleukodystrophy; settling the diagnosis [1].

X-linked adrenoleukodystrophy (X-ALD) is an X-linked recessive disorder affecting approximately 1 in 21,000 males. This disease is caused by defective beta-oxidation of fatty acids in peroxisomes that leads to elevated serum concentrations of very-long-chain saturated fatty acids (VLCFA). The accumulation of cholesterol esters of the fatty acids and gangliosides in the membranes of cells in the brain, adrenal cortex, and other organs causes a primary adrenal insufficiency and progressive neurological dysfunction. The responsible gene, called ABCD1, is located on the long arm of the X chromosome (Xq28), and encodes a peroxisomal membrane protein, called ALDP (adrenoleukodystrophy protein), that belongs to the ABC super family of transporter proteins. Many mutations within ABCD1 have been detected.

There is considerable phenotypic variation among individuals affected with X-ALD. Broadly, cerebral adrenoleukodystrophy begins in childhood and includes behavior disturbances with rapid progression to dementia, blindness, and quadriparesis. Death typically occurs within months to several years of onset of symptoms. Adrenomyeloneuropthy is a milder and more slowly progressing form of X-ALD. It begins in adolescence or adulthood with symptoms of weakness, spasticity, and distal polyneuropathy and may include emotional lability, mania, or psychosis [2,3]. Bladder dysfunction is a common manifestation of adrenomyeloneuropathy and can be a presenting symptom of this disease [4,5].

Males carrying an X-ALD mutation will almost invariably develop disease, with about 50% developing adrenoleukodystrophy in early childhood and 50% developing adrenomyeloneuropathy later in adolescence or adulthood. Both phenotypes occur in the same family [6]. Approximately half of heterozygous female carriers develop an AMN like syndrome [2].

The common concept of adrenoleukodystrophy involves images from the popular film *Lorenzo’s Oil*, which was based on the true story of a young patient with the cerebral form of X-ALD. Although our patient had been specifically concerned about adrenoleukodystrophy, his efforts to be tested were always dismissed because he did not appear extremely ill. The adrenomyeloneuropathy phenotype is not well known, yet, our patient easily fits into its description. Victor, Ropper 2001 report, “We are caring for several adult men (with X-ALD) in whom the cerebral symptoms have been mild, allowing for high level cognitive function, the main manifestation consisting of personality quirks, spastic gait, urinary difficulty, testicular insufficiency, and baldness. Two of the men related the characteristic history of a male sibling who dies in childhood, ostensibly of Addison disease” [7].

X-ALD is estimated to be the cause of adrenal insufficiency in approximately 35% of patients with idiopathic Addison’s disease and should be considered in the differential diagnosis of any male with adrenal insufficiency. Neurological symptoms, such as changes in gait or peripheral neuropathy, may appear before adrenal insufficiency [8]. Patients may also present with psychiatric disturbances months to years before onset of other problems [3].

## Conclusion

Adrenal hormone replacement therapy is critical and may be life saving. Our patient reported a brother that died of influenza at the age of eight. From the history, it seems likely this brother also had X-ALD and was suffering from adrenal insufficiency. An Addisonian crisis in a young male can be an acute presentation of X-ALD.

Data from patients with X-ALD, treated with glycerol trioleate and glycerol trierucate in a 4:1 ratio (Lorenzo’s oil), showed significantly reduced levels of VLCFA’s within four weeks. Also, Lorenzo’s oil is associated with reduced risk of developing MRI abnormalities in asymptomatic boys with X-ALD [2]. Bone marrow transplantation (BMT), and umbilical cord hematopoietic stem cell transplantation (HSCT) are the only proven effective therapy for symptomatic cerebral forms of X-ALD [10].

Early identification of X-ALD, preferably before the onset of symptoms, is critical to maximizing treatment. Identification of hemizygotes can be made by testing levels of VLCFA. These levels are elevated at birth, providing the opportunity for neonatal screening. Although elevated levels of VLCFA are clearly indicative of hemizygosity, only 85% of heterozygous females have abnormal levels. Therefore it is recommended that male relatives in an affected family be tested, even if their mother has normal VLCFA levels [1].

For our patient, testing for high levels of VLCFA within the patient’s pedigree ensued. The patient’s mother, (an obligate carrier,) and one sister had elevated levels of VLCFA associated with heterozygosity. A second sister had normal levels and her three children, all boys, also had normal levels. A third sister had a male child and he tested normally. The mentally retarded brother had normal levels of VLCFA. There were two maternal aunts with normal results, and no maternal uncles. The patient’s brother with ataxia refused testing. This brother had one child, a male, which was determined not to be at risk, due to the X-linked inheritance.

The patient and his heterozygous sister enrolled in a clinical trial of Lorenzo’s Oil at Kennedy Kreiger Institute.

## List of abbreviations

ACTH: Adrenocorticotropic hormone
B12: Cobalamin
DHEA: Dehydroepiandrosterone
HSCT: Hematopoietic stem cell transplantation
MRI: Magnetic resonance imaging
PSA: Prostate-specific antigen
Triple A Syndrome: Achalasia-addisonianism-alacrima syndrome
VLCFA: Very-long-chain saturated fatty acids
X-ALD: X-linked adrenoleukodystrophy
